# Personal

**Published:** 1900-09

**Authors:** 


### PERSONAL.

Dr. Francis E. Fronczak and Mrs. Fronczak have returned from their European tour. They traveled about the continent for four months, as outlined in the itinerary printed in the May issue of the Journal.

Dr. Fronczak spent some time in study in the clinics of Cracow, Lemberg and Warsaw, and took part in the Ninth Congress of Polish physicians and scientists at Cracow, which was attended by over 1,000 members, where he delivered an address on Medicine in America. It was well received and will be printed in full in the Polish medical journals. He also attended the 500th anniversary of the foundation of Cracow University, as the special delegate from the University of Buffalo; other delegates were present from Chicago, Johns Hopkins, Harvard, Catholic University of Washington and Georgetown University. England had representatives from Oxford, Cambridge, Edinburgh, Glasgow and other universities. He attended the Thirteenth International Congress, at Paris, visited the Exposition and has returned with increased physical and mental vigor.

Dr. Fronczak is civil service commissioner of Buffalo and one of our most accomplished Polish physicians.

Dr. Helen J. C. Kuhlman, of Buffalo, assistant physician at the Buffalo State Hospital, was naturalised as an American citizen by County Judge Emery recently. Dr. Kuhlman was born in Germany and has lived in the United States thirteen years. She is the first woman to be naturalised in County Court since Judge Emery came to the bench.

---

Dr. George E. Brewer, U. of B., '84, of New York, has been appointed junior surgeon to Roosevelt Hospital with continuous service. Dr. Brewer was a pupil of the late Prof. Julius F. Miner, of Buffalo, and is gradually and assuredly acquiring a strong position among the foremost surgeons in the metropolis.

---

Dr. Albert H. Macbeth, formerly of Buffalo, and Dr. Harriet Stemen, of Fort Wayne, Ind., were married, July 3, 1900, at the home of the bride's parents, Dr. and Mrs. C. B. Stemen, of Fort Wayne. Dr. Macbeth's many Buffalo friends will unite in congratulations upon this joyous event.

---

Dr. M. B. Searls, U. of B., '69, has recently been re-elected a member of the school board of East Aurora. Dr. Searls has proven a good officer and his return to the board for another term is a proper recognition of his services.

---

Dr. F. G. Haines, of Warren, Pa., spent two weeks in Buffalo during the month of August, 1900, visiting the several hospitals of the city, studying surgical technique. He was much pleased with his visit.

---

Dr M. F. Clausius, U. of B., '91, of Barrington, Ill., has been appointed acting assistant surgeon U. S. Army, and expects to leave for his post of duty in the Philippines early in September.

---

Dr. George N. Jack, U. of B., '95, was elected a member of the school board of Depew, at the recent annual meeting. This is a good selection and means progress in our new suburb.

---

Dr. Eugene A. Smith, of Buffalo, has removed his office and residence from 831 Ellicott Street to 1018 Main Street. Hours: 1.30 to 3.30 p.m. Telephone, Tupper 342.

Dr. Sidney A. Dunham, of Buffalo, is spending a few weeks in New York, studying diseases of the nervous system under the direction of Dr. Frederick Peterson.

Dr. Frederick W. Love, of Buffalo, has been appointed physician of the fourth health district to fill a vacancy caused by the death of Dr. G. W. Lewis.

Dr. H. T. Williams, of Rochester, has removed his office and residence from 52 Clinton Place, to 274 Alexander Street. Telephone 338.

